# Supplementary material for: Prevalence and risk factors of childhood diarrhea among wastewater irrigating urban farming households in Addis Ababa
Source: PLoS One. 2023 Nov 8;18(11):e0288425. doi: 10.1371/journal.pone.0288425 (PMC10631672; doi:10.1371/journal.pone.0288425)
Supplement: Attachment — Submitted filename: Response to the comments.docx [file pone.0288425.s002.docx]

Response to comments

This letter contains three parts

- A. Revisions made as per journal requirements

- B. Responses given to the academic editor’s comments

- C. Responses given to the reviewers’ comments

A. Journal requirements

1. PLOS ONE - Style requirements

- File naming are modified following the PLOS ONE’s style

2. Professional language editors (two of the authors are native English speaker, from Newcastle University, UK.

- Dr Claire Walsh – Co-author

[School of Engineering, Newcastle University (UK)](https://www.ncl.ac.uk/engineering/staff/profile/clairewalsh.html), Email: [claire.walsh@ncl.ac.uk](mailto:claire.walsh@ncl.ac.uk)

Telephone: +44 (0)191 208 6647, Twitter: [@clwnewc](https://twitter.com/clwnewc)

<https://www.ncl.ac.uk/engineering/staff/profile/clairewalsh.html>

- Dr. Michaela Goodson – Co-author

Email: [michaela.goodson@newcastle.edu.my](mailto:michaela.goodson@newcastle.edu.my)

Address: Newcastle University Malaysia
Jalan Sarjana 1, Educity@Iskandar
Iskandar Puteri, Johor, 79200

<https://www.ncl.ac.uk/numed/people/staff-profiles/michaelagoodson.html>

3. ORCID iD for the corresponding author

I updated my profile information in the editorial manager. <https://orcid.org/my-orcid?orcid=0000-0003-1962-1774>

4. Your ethics statement should only appear in the Methods section of your manuscript.

Ethical statement is included at the end of methods part, just after data analysis section.

5. We note that Figure 1 in your submission contain [map/satellite] images which may be copyrighted.

Figure 1, map of the study area is the map of the 10 sub-cities of Addis Ababa with overlaying rivers (SRTM 30x30 ArchGIS 10.8). We generated the map by GIS Software using map data and shapefiles within the public domain and with open access licenses from <https://www.usgs.gov/centers/eros/science/usgs-eros-archive-digital-elevation-shuttle-radar-topography-mission-srtm-non>

www.usgs.gov (<https://www.usgs.gov/centers/eros/science/usgs-eros-archive-digital-elevation-shuttle-radar-topography-mission-srtm-non>)

B. Response to the editor

Dear Editor, first up, we express appreciation to your timely processing of our manuscript and your comments. We seriously considered yours and the reviewers’ comments. We hope that we addressed to all the clarifications, questions and comments forwarded in a given timeframe. We kindly request you to see the detail responses for your ‘5’ issues are answered together with the reviewers’ comments.

1. [Issue 1, e.g. Clarification of the research question and better alignment of the objectives with the methodology]

Clarification is given in the introduction and methodology part. The research questions/problem statements are elaborated rewriting in an improved way and adding more points.

2. [Issue 2, e.g. Expansion of the literature review to address earlier relevant studies and establish the context for your work]

We revised the introduction part as per the comments. Furthermore, additional references/earlier works in related areas are used for elaboration.

3. [Issue 3, e.g. In-depth description of the data collection and analysis procedures to ensure reproducibility]

Based on the comments and suggestions given, the methodology part including the study design, sample size determination and sampling techniques are revised accordingly.

4. [Issue 4, e.g. Consideration of alternative explanations for the findings and a more nuanced interpretation of the results]

Parts of the finding and interpretation which was not clear; and need better clarification, we made revisions.

5. [Issue 5, e.g. Improvement of the manuscript's structure, organization, and clarity]
We made internal structural changes of the manuscript. We clarified unclear and less explained statements and ideas throughout the text.
C. Response to Reviewers

Response to reviewer #1

Thank you, dear reviewer. Giving fully consideration, we tried to respond to your comments one by one.

Material and Method

The sanitary situation of the study area (lines 105–111) may be deleted, and this can be discussed in the discussion

We shifted it to the discussion part accordingly.

There is no sampling technique used to select the households.

Sampling technique is included in section 2.2.

It is mentioned that the sample size was determined based on a ‘longitudinal study designed to investigate the incidence of diarrhea’, but the title and objective of the study specify that the prevalence of diarrhoea would be investigated; this is inconsistent and the study is not a longitudinal study.

Correction is given. At the beginning, the sample size was determined for a longitudinal study, but later on, we changed it to a cross-sectional study. Now, we took the correction accordingly.

This study's design is also not mentioned explicitly.

- Now in this revision, we further elaborated the design at sections 2.2 and 2.3

Furthermore, it is unclear how the researcher knew, prior to data collection, that the selected households had children under the age of 5. It appears that the questionnaire was pretested on the same population, which is not appropriate.

- We used an inclusion - exclusion criteria such as “only wastewater-irrigating farming households”, “only farming households having children under the age of 5 are included”. Thus, we included only those households who have children under the age of 5 are included in the study. The tools were pre-tested on those farming households who were not selected by simple random sampling.

Results: Out of the 402 sample size, 386 responded; however, the total number of participants in some variables is different and inconsistent with the responded size in tables 1 and 2.

- Yes, we corrected it. The major difference was typographical errors. Some missed values may also be in some variables, but statistically considered during analysis.

- We corrected all P-values to be written consistently.

It is preferable to write ‘p<0,05’ rather than ‘P<0.05’.

- Thank you very much for your comments. I think both ways are possible but I checked the PLOS ONE journal style, it follows the full-stop style, i.e. p<0.05 rather than p<0,05

In the results section, only findings should be given; there should be no discussion or citation of other studies.

- We revised it accordingly.

It is not clear how many under-5 children suffered from diarrhea out of the total number children studied in the previous two weeks.

We revised it. We added a statement in the prevalence section, which an equivalent number for the 22.3% of the participants, i.e. approximately 86 children.
Conclusion: The statement in lines 355–356 is not supported by the findings.

- We revised the statement.

Response to reviewer #2
Thank you, dear reviewer. Giving fully consideration, we hope that we addressed to all your clarifications and comments one by one.

Abstract
Please specify the method that you used for respondent selection

We revised it by adding specific approaches we applied during study population selection.
Introduction
Add the implication of your study to international readers and future research.

We added an elaboration as per the comments given.
Are the people in Addis Ababa drink from irrigation system?

- No, the river is severely polluted. However, those farming households along the rivers in addition to using the wastewater for irrigation, they use it for cloth washing, for bathing, washing vegetables and livestock drinking.

Is the wastewater-contaminated river water as the primary source of drinking water?

- No, it is not source of drinking water.

How the irrigation water become the risk factor of diarrhea in that place?

Wastewater pathogens can reintroduced into the households in several ways including washing bodies, clothes and vegetables with the irrigation water, cloth contamination, using working clothes at home, and bringing farm tools to home without onsite washing. Once the pathogens reach to the domestic environment, it can be ingested directly from hand to mouth, through food, plate and drinking water contamination and soon. Children at home touch the contaminated clothes, boots, farm materials and can contract the pathogens.

Are people of Addis Ababa consumed raw vegetable from farming area that used wastewater irrigation? You should elaborate this information in introduction.

Yes. The wastewater irrigation supplies more than 90% of the leafy vegetables and more than 65% of all types of vegetables. Those leafy vegetables such as lettuce and chard, which normally eaten raw, are produced from the wastewater irrigation and sold everywhere in the city.
Add the previous study that claimed there are microbiological contamination in vegetable or drinking water sources in addis ababa.

We added literatures related with this part.

How many diarrhea cases in your study area?

We included in section 3.2.
Inform the reader, your study respondent and why?
We revised the problem statement part in the introduction section.

Material and method
why you only choose two rivers (Big Akaki and little Akaki) as your study site?

They are the only rivers in the city, cross the city from north to south. They receive all types of pollutants from multiple sources including households, hotels, restaurants, government institutes, industries, hospitals and other health service sectors. Although the rivers are severely polluted, they are intensively used for vegetable production, livestock drinking, cloth washing, construction etc. Therefore, they are becoming a threat to the public health.

Attach structured questionnaire in supplementary material section.

The questionnaire can be given as supplementary material

Is there any Inclusion and exclusion criteria for your respondents?

Yes, we have. Now, in this revised version we added inclusion criteria at section 2.2

Make sure you have number registration for STATA software.

Yes, I was using licensed software.

Result and discussion
What are protective wears for farmworker that you mean in the result?

Protective wears include those wears protecting the farm workers’ body and clothes from wastewater during occupation. These include boots, plastic protective coating, masks etc.

Why it is influence diarrhea cases in Addis Ababa?

The third highest diarrhea prevalence in the country is found in Addis Ababa. WASH condition in Addis Ababa is very poor. Sanitation condition is very poor (indicated in the discussion part). There is no full water supply coverage in the city, particularly in the slum areas. Almost all the city water supply is intermittent. These conditions are usually associated with increased occurrence of diarrhea.

How children are exposed to diarrhea? Add some citation from the previous studies to discussion section!

We revised accordingly. Included in the discussion part.
Why walking through the farm during irrigation will influence diarrhea cases in children.

During irrigation activity, most farmers without any protective wears walk across the farm to channelize the irrigation water, which causes direct contamination of the foot and clothes. Moreover, walking through splashes the irrigation water and contaminate the farmers’ cloth. There is no onsite body washing practice in all of the farm workers, which implies that the farmers go home with their contaminated body and clothing and reintroduce wastewater pathogens to domestic environment, where children normally touch and manipulate everything.

Based on your study result, what are your recommendation to prevent diarrhea?

Our recommendation spans around awareness creation/ education and improving WASH conditions among the farming households.

What your study implication for further research?

As a way forward, ways to reduce wastewater-related pathogen loads such as by employing onsite wastewater treatment need to be considered.

Please make sure your bibliography style in accordance with PLOSone journal style!

We changed the bibliography from ‘numbered’ style to ‘Vancouver superscript bracket’ style.
