# Supplementary material for: Prevalence and risk factors of childhood diarrhea among wastewater irrigating urban farming households in Addis Ababa
Source: PLoS One. 2023 Nov 8;18(11):e0288425. doi: 10.1371/journal.pone.0288425 (PMC10631672; doi:10.1371/journal.pone.0288425)
Supplement: Attachment — Submitted filename: Response to Reviewers comments.docx [file pone.0288425.s003.docx]

**Response Reviewer #1**

*PONE-D-23-03536R1: Prevalence and Risk Factors of Childhood Diarrhea among Wastewater Irrigating Urban Farming Households in Addis Ababa*.

Dear reviewer, thank you for your valuable comments, which significantly improved the quality of our manuscript.

**Reviewer #1**

Minor changes are required to the updated document.
The author(s) revised ‘p<0.05’ but some ‘P<0.05’ yet to be revised.
In the text, the reference number should be cited sequentially, such as [6].

- We made necessary revisions as per the comments given by changing “P<0.05” to “p<0.05”.
- References numbers are updated and corrected throughout the paper.

**Materials and Methods:**

It is stated that twelve urban framing sites were chosen for data collection. It should be mentioned that how many of the twelve urban framing sites, were wastewater-irrigated farming sites and how many were non-wastewater-irrigated farming sites?

- Now, we specified and separately mentioned the two groups.

The calculated sample size was 407, although it is mentioned that data was collected from 402 households, with responses obtained from 386 of them.
**-** We corrected the typographic error in sample size.

**Results**

In the title of Table 1, ‘occurrence of diarrhea’ should be included.

- We revised the table as per the comments given.

In the tables, the number of responses against each variable is not comparable; therefore, it is preferable to mention the total number of responses against each variable.
 -	Now, the error is corrected.

**Conclusion**

It was indicated that the households may be exposed to toxic chemicals; this should be discussed in the discussion before making any comments in the conclusion.

- We made necessary amendments in the conclusion part.
